# Implications of time and space factors related with youth substance use prevention: a conceptual review and case study of the Icelandic Prevention Model being implemented in the context of the COVID-19 pandemic

**DOI:** 10.1080/17482631.2022.2149097

**Published:** 2022-11-23

**Authors:** Tanya Halsall, Kianna Mahmoud, Srividya N. Iyer, Heather Orpana, Megan Zeni, Kimberly Matheson

**Affiliations:** a Youth Research Unit, University of Ottawa Institute of Mental Health Research at The Royal, Ottawa, Ontario, Canada; b Department of Neuroscience, Carleton University, Ottawa, Ontario, Canada; c Faculty of Medicine, Dalhousie University, Halifax, Nova Scotia, Canada; d Department of Psychiatry, McGill University, Montréal, Québec, Canada; e Centre for Surveillance and Applied Research, Public Health Agency of Canada, Ottawa, Ontario, Canada; f Faculty of Education, University of British Columbia, Vancouver, British Columbia, Canada

**Keywords:** Substance use prevention, COVID-19 pandemic, Icelandic Prevention Model, bioecological model, case study, qualitative

## Abstract

Purpose: This research examines the implementation of the Icelandic Prevention Model (IPM) in Canada to identify opportunities revealed by the COVID-19 pandemic to re-design our social eco-system to promote wellbeing. This paper has two objectives: 1) to provide a conceptual review of research that applies the bioecological model to youth substance use prevention with a focus on the concepts of time and physical space use and 2) to describe a case study that examines the implementation of the IPM in Canada within the context of the COVID-19 pandemic. Method: Study data were collected through semi-structured qualitative interviews with key stakeholders involved in implementing the IPM.

Results: Findings are organized within three over-arching themes derived from a thematic analysis: 1) Issues that influence time and space use patterns and youth substance use, 2) Family and community cohesion and influences on developmental context and time use and 3) Opportunities presented by the pandemic that can promote youth wellbeing.

Conclusion: We apply the findings to research on the IPM as well as the pandemic to examine opportunities that may support primary prevention and overall youth wellbeing. We use the concepts of time and space as a foundation to discuss implications for policy and practice going forward.

## Conceptual review

The COVID-19 pandemic that emerged in the spring of 2020 resulted in rapid social change and forced us to reconstruct how we live on a global scale. Many of these changes moved us outside of commonly held assumptions with respect to social and health policy and had a significant influence on our time use as well as movement patterns. In Canada, the pandemic has had a negative impact on child health and wellbeing (Cost, 2021), has been associated with an increase in substance use among some populations (MacEachern et al., 2021), and has resulted in a substantial worsening of the opioid overdose crisis (Special Advisory Committee on the Epidemic of Opioid Overdoses, 2021). However, the pandemic also presents an opportunity to re-conceptualize policy to support social justice and improve child health and wellbeing (Russell & Stenning, 2020). Further, some of the lifestyle adjustments resulting from pandemic public health measures align with factors that promote healthy child development and that may prevent substance use behaviours. These factors include increased time with family (Evans et al., 2020; Pelletier et al., 2021; Williams et al., 2021), increased interaction among community members (Innocent & Stevens, 2021; Russell & Stenning, 2020) and a reduction in stress related to the fast pace of life and pressures from school (Cornell, 2021; Hawke et al., 2020; Power, 2020). Further, public health restrictions may have reduced exposure to some social influences that encourage substance use (Thorisdottir et al., 2021).

The path to “build back better” (Government of Canada, 2021b) necessitates the examination of new opportunities revealed by the pandemic and to leverage them to re-design our social eco-system to promote wellbeing for all. This paper has two goals. First, it presents a review of research related to the bioecological model (Bronfenbrenner & Morris, 2006) that highlights the concepts of time and space to examine risk and protective factors that influence substance use in young people. Second, it presents findings from a larger case study examining the implementation of the Icelandic Prevention Model (IPM; Halsall et al., 2020) within a rural community in Canada that explores issues related with time and space use and how they influence youth development and substance use behaviours. These findings also offer a glimpse into the realities of the community experience of the pandemic and perceptions of the related impacts that may influence youth and community wellbeing in the future.

### The bioecological model

The bioecological model is a theory designed to understand human development and consists of four components: 1) process, 2) person, 3) context, and 4) time (Bronfenbrenner, 1995, 1999; Bronfenbrenner & Morris, 2006). The *process* component is a fundamental concept that represents the interactions between a developing individual and the people, objects and symbols within their immediate surroundings. The model suggests that these interactions are the main drivers of change and development and must be present over a minimum duration and frequency to exert their influence. Depending on the nature of the interactions, they may drive individual functioning towards a positive or negative direction (Bronfenbrenner & Morris, 2006). Within the model, the *person* represents the characteristics of the developing individual, including disposition, resources (skills or abilities) and demand characteristics that elicit reactions from the social context that influence development. *Context* is divided into micro-, meso-, exo- and macro-systems that represent nested environmental systems that influence development (Bronfenbrenner, 1979; Bronfenbrenner & Morris, 2006). Microsystems refer to the contexts where a developing individual is physically present, such as the home, school and neighbourhood. The mesosystem is defined as “the inter-relations among two or more settings in which the developing person actively participates (such as, for a child, the relations among home, school, and neighborhood peer group; for an adult, among family, work, and social life). A mesosystem is thus a system of microsystems” (Bronfenbrenner, 1979, p. 25). Bronfenbrenner hypothesized that developmental contexts have a greater potential for positive influence when there are more supportive links between them. Developmental potential is enhanced when the linkages are created by significant others (for example, a parent or caregiver). Exo-systems are contexts that have an influence on development factors, such as a school board or municipal governing body, where the developing individual does not actively participate. The macro-system represents social and cultural norms that influence the characteristics of the other developmental systems (Bronfenbrenner, 1979; Tudge et al., 2009).

*Time* is a more recent element of the theory (Bronfenbrenner & Morris, 2006; Tudge et al., 2016). The field has generally placed an overemphasis on the contextual aspects of the theory (Bronfenbrenner, 1986; Bronfenbrenner & Morris, 2006; Jaeger, 2016), therefore, the concept of time has not received a significant amount of attention in the literature. However, time is of key significance as a developmental context and the related proximal processes can only convey their influence through exposure over a considerable quantity of time (Bronfenbrenner & Morris, 2006). Yet, this has often been ignored within practice, wherein short-term programming that places the onus of achieving wellness of the individual are favoured over system-level initiatives that might have more substantive influence through developmental contexts (Adler & Stewart, 2009; Patel et al., 2018; Ungar, 2005). “Young people do not grow up in programs, but in families, schools, and neighborhoods” (Roth & Brooks-Gunn, 2003, p. 97). Therefore, interventions should place a stronger emphasis on the social and physical environments where individuals spend the majority of their time.

In Canada, the prevalence of any alcohol use in the past 12 months among students from grade seven to 12 is 44% and it is 18% for any cannabis use (Health Canada, 2019). Several elements of the bioecological model are strongly implicated in youth substance use behaviours and are important to consider when designing interventions. These include, among others, social norms (macro-system), personal dispositions (person) as well as time use patterns (time). The majority of adolescent drinking behaviour is motivated by social reasons (Kuntsche et al., 2005) and social norms of substance use in adolescence have been found to predict drinking and other substance use behaviour (Brooks-Russell et al., 2014; Eisenberg et al., 2014). There are several personality traits that have been identified as being most strongly associated with excessive drinking, including sensation-seeking and impulsivity, extraversion, low conscientiousness, anxiety sensitivity and neuroticism (Adan et al., 2017). Further, brain development during adolescence increases individual predisposition for risk-taking (Patel et al., 2018) while self-regulation skills are still developing (Arain et al., 2013). In contrast, higher levels of relatedness and feelings of competence among youth are associated with a lower odds of cannabis use, frequent cannabis use, alcohol use, and binge drinking (Enns & Orpana, 2020). In the next section, we review relevant research that examines the relationship between time use patterns and substance use as well as key issues that can inform prevention.

### Time use patterns and substance use

Time use patterns have been explored within youth development literature, particularly in examinations of extracurricular time, and many studies have identified that how time is spent can have an influence on developmental outcomes (Benson, 1997; Larson, 2000; Mahoney et al., 2003; Shanahan & Flaherty, 2001). Rose-Krasnor (2009) highlights the need for the field to further consider intensity and duration dimensions of activity involvement as well as the potential influence of high-density activities, such as residential camps and retreats. Constructive use of time has been identified as a developmental asset associated with better developmental outcomes, together with a range of individual characteristics and contextual factors (Benson, 1997). Constructive use of time is characterized by time spent in activities such as sports, creative pursuits, homework, volunteering and extracurricular activities. There is also a robust literature that has identified that unstructured time spent with peers in the absence of adult supervision has been associated with involvement in substance use (Hoeben et al., 2016; Meldrum & Leimberg, 2018; Osgood et al., 1996).

Paradoxically, there is emerging evidence that an over-abundance of adult supervision, structured time, and lack of opportunity for self-directed outdoor play can have a negative impact on the health and wellbeing of younger children. The Position Statement on Active Outdoor Play (Tremblay et al., 2015) was released in response to building evidence that children are missing out on important health and developmental opportunities as a result of increasing risk aversion in relation to activities out of the home, and of time spent on screens. Outdoor unstructured play can enhance levels of physical activity, mental health, cognitive development and opportunities to create social connections (Tremblay et al., 2015). A key developmental opportunity that is provided by outdoor play is engagement in risky play, which includes activities that involve elevated height/speed, dangerous features or instruments, or rough play. (Sandseter, 2009). Risky play is defined as thrilling or exciting play that includes an uncertainty of outcome or physical injury. (Brussoni et al., 2015; Sandseter, 2009; Sandseter & Kennair, 2011). Another activity that is related with risky play is independent mobility. This term is used to describe children’s movement within their community without adult supervision, often involving active transportation to various destinations (e.g., walking/biking to school or a park; Marzi & Reimers, 2018). Similar to other outdoor play activities, independent mobility has declined over recent years (Brussoni et al., 2015; Pelletier et al., 2021; Tremblay et al., 2015). In a systematic review (Brussoni et al., 2015), risky play was found to be associated with increased physical activity, healthier weight and motor skills, improved mental health, including enhanced competence and self-esteem, and stronger social skills, such as ability to manage conflict and greater independence. Of key importance, participation in risky play may support the development of risk management strategies and an enhanced capacity to navigate risks encountered in adolescence such as substance use (Ungar, 2007). Relatedly, Hansen examined the trait of sensation seeking in adolescents in relation with positive (e.g., adventure sport) and negative (e.g., substance use) risk-taking as well as the level of opportunities provided from the family and school contexts to participate in challenging activities (Hansen & Breivik, 2001). They found that sensation-seeking was associated with both positive and negative risk behaviours, but that young people who did not have any opportunities to experience challenges were more likely to participate in negative risk-taking behaviour. To resolve these conflicting bodies of evidence with respect to time use patterns, it will be important to examine interventions that influence developmental environments and opportunities for structured and unstructured activities through the course of development. In the next section, we examine literature that identifies how the pandemic has influenced time use patterns of children and youth. Further, we introduce the Icelandic Prevention Model (IPM) as a prototype intervention designed to influence developmental contexts and time use patterns to reduce involvement in substance use.

### The pandemic and the Icelandic Prevention Model (IPM)

The pandemic and related restrictions to protect health have had a significant influence on the nature of our daily activities, as well as our use of time. In Ontario, public health policies that were developed to mitigate harms from the coronavirus evolved as the pandemic progressed and new evidence emerged. During the first and second waves of the pandemic, which coincided with this data collection, public health measures included workplace closure (with the exception of those deemed essential services), school closures, restrictions on gathering size, mask requirements, closure of public transit and stay at home requirements, among others (CBC, 2020; Ministry of Health, 2020; Ontario Newsroom, 2020).

Associated with these changes was a significant range of detrimental effects on children, youth and families, including reduced levels of physical activity (Mitra et al., 2020; Moore et al., 2020), time out of school, reduced contact with peers (Ellis et al., 2020), and increased depression and anxiety (Hawke et al., 2020). However, there were several key shifts that resulted from the pandemic that had implications for youths’ time use, substance use behaviours and wellbeing (see Vaillancourt, Brittain, Krygsman, Farrell, Landon, & Pepler, 2021; Hawke et al., 2020; Pelletier et al., 2021; Russell & Stenning, 2020; Thorisdottir et al., 2021). For example, research that was conducted during lockdowns has identified that substance use behaviours decreased in young people in Canada and Iceland (Hawke, 2020; Thorisdottir et al., 2021). Further, work-from-home conditions paired with the closure of recreational activities supported an increase in unstructured play and independent mobility in younger children (Pelletier et al., 2021). Physical distancing measures created conditions where individuals spent more time outdoors and in their communities creating opportunities to enhance community connection (Innocent & Stevens, 2021) and make neighbourhoods safer (McMillen et al., 2019; Rydin et al., 2012; Saville, 2009). Many social and recreational activities were moved outdoors (Weinbrenner et al., 2021; Williams et al., 2021) and this supported increased interaction with neighbours (Innocent & Stevens, 2021; Russell & Stenning) as well as increased opportunities for children’s outdoor play (Pelletier et al.; Szpunar et al., 2021). These behaviours strengthen relationships among community members:

We know that children’s play is at the heart of healthy communities, for children themselves, for their families, and often for others too … Play can be seen as a catalyst for community, through the importance of space, trust, freedom and, often, intergenerationality. In the context of COVID-19, neighbourhoods have become key sites of support and will need to continue to be for some time …. (Russell & Stenning, 2020, p. 2)

Further, recent changes to policy created some new opportunities for family supports that may promote youth development, including increased flexibility in workplace policies (CBC, 2020a; Mehdi & Morisette, 2021), enhanced investments in daycare (Prime Minister of Canada, 2021) and provincial program for paid sick leave (Ontario, 2021).

Some of the new developments emerging from the pandemic, such as policy supports and enhanced community cohesion, overlap with the kinds of strategies that are implemented within the IPM. The IPM is a community-based participatory approach that is designed to support upstream prevention of substance use in youth. The strategy engages community leaders to develop tailored interventions that address risk and protective factors within the family, peer, school and community contexts (Kristjánsson et al., 2020a, 2020b). Fundamentally, the model is designed to enhance the social environment with the objective of reducing youth substance use behaviours by making “the healthier choice the easier choice” (World Health Organization, 1986, p. 2). The model is based on five guiding principles and ten core steps that guide implementation within an iterative approach to support long-term community development (see, Table I). Community strategies are identified through a data-driven approach that target identified issues. Examples of strategies that have been used in Iceland include the development of leisure cards to support population-wide participation in extra-curricular activity, parental contracts to promote community safety and policy restrictions to reduce substance use.

**Table I.** The five guiding principles and ten core steps of the Icelandic Prevention Model.

	Guiding Principles
Principle 1	apply a primary prevention approach
Principle 2	engage community action and public school involvement
Principle 3	engage stakeholders using high-quality data
Principle 4	integrate researchers, policy makers, practitioners, and community members
Principle 5	align the scope of the solution with the nature of the problem
	Ten Core Steps
Step 1	Develop local coalition and capacity building
Step 2	Identify local funding and capacity building
Step 3	Community engagement and pre–data collection planning
Step 4	Collection of population-level data regarding youth substance use behaviour, risk and protective factors, including data-driven diagnostics
Step 5	Enhance community engagement
Step 6	Disseminate survey findings
Step 7	Community-driven goal-setting based on survey findings
Step 8	Align policy and practice with community goals
Step 9	Children and adolescents are exposed to healthier developmental contexts
Step 10	Repeat previous steps annually or bi-annually

The IPM aligns with the principles of the bioecological model in that it takes a comprehensive approach that involves a range of community stakeholders to enhance developmental contexts in order to reduce the risk of youth substance use. Time use patterns are a significant focus and interventions are designed to increase availability of extracurricular activities that support constructive use of time and decrease unstructured unsupervised time. Finally, many previous implementation strategies have included interventions that are designed to increase linkages between microsystems and effectively enhance mesosystems. For example, previous strategies that enhance linkages among microsystems include strengthening connections among families, between families and schools and between parents/caregivers and peers.

In some respect, the pandemic has impacted social norms in ways that have replicated the community intervention strategies that are implemented within the IPM, such as decreased unstructured, unsupervised time for youth, increased time with family and increased community connections. This research concurrently examines community member perceptions of the implementation of the IPM as well as their experiences of the pandemic. It also highlights alignment between social influences and opportunities to leverage these impacts to support youth development and wellbeing.

## Case study of the Icelandic Prevention Model

Given the contrasting bodies of evidence that highlight the beneficial impact of constructive use of time for adolescents and need for more unstructured unsupervised time for children’s play, it is important to examine time use issues more comprehensively in relation to development and how they might influence substance use in youth. Further, since the pandemic has had such a pervasive impact on lifestyle and creates potential opportunities for influence on time use and movement patterns, it is important to examine these issues in the context of the pandemic.

Lanark County is the first community in Canada to implement the IPM as Planet Youth Lanark County (PYLC). Lanark County is populated by approximately 75,625 people across 3,036 km2 in southeastern Ontario. It is made up of nine municipalities that include a combination of rural and a few more densely populated communities. The school-wide survey (Step 4 of the IPM) was collected from 70% of all grade tens within the two English-language school boards in the region (N = 497) in February 2022. 30% of respondents had gotten drunk at least once, 12% had been drunk in the last 30 days, 33% had tried alcohol before the age of 13 and 21% had tried cannabis (Planet Youth Lanark County, 2022). PYLC is currently being led by the PYLC Steering Committee and at the time of publication, they had completed the first six steps of IPM implementation. PYLC was formed in response to community concerns regarding the opioid crisis (see Halsall, Mahmoud, Pouliot, & Iyer, forthcoming). Activities within the first six steps include the creation of a local coalition, development of community engagement, funding and support, preparation for the collection of a population-level youth survey, data collection, community engagement and dissemination of the findings (see, Kristjánsson et al., 2020b). In this paper, we describe findings from a case study designed to examine the implementation of PYLC and explore issues related with time and space use as well as how this affects youth substance use. In addition, we examine community experiences of the pandemic and participant perceptions of relevant implications on youth wellbeing.

### Methods

This study is a component of a larger mixed methods case study that was guided by a pragmatic research paradigm and designed to examine the implementation and impact of the IPM within Lanark County (see Halsall et al., 2020; Halsall, et al., forthcoming). Data were collected through semi-structured qualitative interviews with PYLC Steering Committee members. The PYLC Steering Committee is made of key stakeholders from across Lanark County who are involved in youth services, including representatives from the schools, public health, justice, faith institutions, social and mental health services, as well as local volunteers. Interviews were completed in the summer and fall of 2020. All PYLC Steering Committee members were invited to participate and interviews were conducted with nine (five men and four women) of about 12 committee members who participated on a regular basis. Many committee members had become involved because of previous work on Municipal Drug Strategy Committees, political experience, leadership within education and lived experience of friends and family who have been affected by substance use issues.

Eight interviews were conducted over the phone and one was conducted through Zoom. Interviews lasted for about an hour (39–95 minutes). Since the overall study was focused on examining implementation processes and outcomes related to the IPM, interview questions were focused on contextual issues, lessons learned, and processes of development and were based on the Quality Implementation Framework (Meyers et al., 2012; e.g., What problems or conditions will PYLC address? Are there any key lessons learned that you would like to share about this process? Please describe.) Interviews were recorded and transcribed using Otter.ai. Transcripts were shared with each participant. Two participants reviewed and approved their transcripts with no revisions or additions.

A thematic analysis (Braun & Clarke, 2006, 2014) was applied by two coders using QSR NVivo. The first coder (TH) has been actively involved in PYLC Steering Committee meetings since the spring of 2019 as a researcher and evaluator. The second coder (KM) became the founding Youth Advisor for the PYLC Steering Committee in May 2021. She grew up in Lanark County and also attended secondary school there before leaving for university. The first author reviewed all transcripts and developed initial codes based on concepts drawn from the bioecological model, community-based health promotion and exploratory categories that emerged from the data. The second author reviewed the initial codes and the two coders met to discuss disagreements and refine codes and over-arching themes. The two coders were in full agreement on the final categories. For a full description of the methods and procedures, please see Halsall and colleagues, 2020. Informed consent was received from all participants. This study was approved by the Royal Ottawa Health Care Group Research Ethics Board and this research meets each of the 21 criteria of the Standards for Reporting Qualitative Research (O’Brien et al., 2014).

### Results

In this paper, we discuss three over-arching themes derived from the analysis of the interview data: Issues that influence time and space use patterns and youth substance use, Family and community cohesion and influences on developmental context and time use and Opportunities presented by the pandemic that can promote youth wellbeing. The first theme is divided into two sub-codes: Lack of access to extracurriculars and over-abundance of unstructured unsupervised time and Challenges related to transportation and accessibility of services.

#### Issues that influence time and space use patterns and youth substance use behaviours

The first over-arching theme is centred around issues that were perceived to influence youth time use patterns that might create circumstances that lead to risky behaviours such as substance use. In Lanark County, some of these issues are created by the distribution of homes across large areas of the community which, in turn, influences parent and caregiver commute times and availability. Participants perceived that this leads to an excess of time young people spend in unstructured and unsupervised activities. Further, geographic spread influences the distribution of infrastructure and programming that can be accessed by young people for extracurricular activities. The lack of after school programming options further exacerbates issues related to unstructured time and youth substance use.

#### Lack of access to extracurriculars and over-abundance of unstructured unsupervised time

Steering committee members talked about the challenges with the gap in afterschool time, the lack of available activities for young people and the need to find ways to structure their time.

I think that one of the big issues that a community, a rural community, or community like Lanark County, which has some kind of, not urban, but some town centres, like [several communities]. And then it has large outlying areas where people are living in the country, in homes that are along country roads, often on farms, but sometimes in rural subdivisions, where the young people cannot participate in any activities without being driven. Whether that is being picked up and driven on a school bus, or their parents driving them. And that’s a big issue. Because the services that are provided, tend to be provided in the towns. (SC 8)

At first it was a sort of a, you know, kids get out of school this early. Their parents aren’t home. They have lots of free time … we need to do something to structure that time and it was probably maybe a third or fourth meeting perhaps that people started talking about surely to God someone’s done something like this somewhere else in the world. (SC 3)

Participants also talked about how many community members were not aware that school finished earlier than before and that some community partners have been trying to fill this gap.

I went to school, many years ago. There was a late bus, 4 days out of 5, I was probably on the late bus, that left like an hour and a half later after school ended. So, you know, because I was going to whatever club I was going to, whether it was the drama club or you know sports, soccer or whatever. [Now], that’s not a possibility. If your parents can’t come and pick you up, you have to get on the school bus. And the other thing is, the school schedule has changed, you know, it’s much earlier. The classic example is, at one of the Youth Centres, one of the board members asked me “Why are the kids coming through the door at 2:20 coming to the youth centre”? And the executive director said “Well, school’s over.” And he says, “Well, that’s ridiculous. School can’t be over.” and she says, “Yeah, that is, school is over.” So, there’s a whole generation of people that, you know, don’t even know that school ends at 2:15 or 2:30 and the kids are out and what do you do? And that’s why the youth centres have tried to fill that gap. (SC 2)

Participants noted that this over-abundance of free time can lead to boredom and engagement in risky behaviour, *“It’s the same issue, you know, kids have the same issues, no matter where they are. Boredom leads to issues and problems.” (SC 2)*

[There was] concern about the extent to which the needs of youth were not being adequately addressed outside of school and the impact that that was having on their either existing or potential willingness to substance use, as a way of turning to abuse. (SC 7)

##### Challenges related to transportation and accessibility of services

Steering committee members identified that there was a need for better transportation to make opportunities (employment, education, extracurricular activities, healthcare and other community assets) more accessible to young people who might be living in a rural context. “There are certainly obstacles when we consider how spread out we are. We aren’t exactly densely populated. Transportation can be an issue.” (SC 7) “When cities want to address things … sometimes it’s a lot more simple … We’ve got a great new center, we’ve got bus passes, people will get to it. It’s all good.” (SC 6) One participant described how their family moved to an urban area after living in a rural community for seven years. This change meant that their child was able to take advantage of better public transportation and recognized the contrast of living in a rural location that lacked transportation. Their child described their relative isolation while living in a rural area as feeling like being grounded:

We moved when [my son] was going from grade 11 to grade 12. And, and so we moved into the city and he can take city transportation and he could stay after school, hang out with his friends join a club, he didn’t have to catch a bus to take him out, back out to [Community]. I wasn’t having to leave work to drive and pick him up and drive him home … And that was that’s true for all children and young people who live in the country. And in his case, he chose to frame it as being grounded for seven years. But that makes the point, right? (SC 8)

One Steering Committee member also noted that the rural context can make it more challenging to dissuade problematic substance use behaviour since it is not as feasible to have a community presence in isolated areas.

The difference between a rural and urban setting, in a rural setting, it can be hidden. Drinking can be hidden much easier. You know, a bunch of kids can go into a forest area and they can drink and the chances of the police driving by and finding them or seeing them are much slimmer than in Ottawa, for example. I mean, there’s more eyes around. Kids go and find an old house or barn that’s not being used, they get a group and the next thing you know, they have a barn [party] and you have 40 or 50 kids show up. (SC 2)

#### Family and community cohesion and influences on developmental context and time use

The Steering Committee members talked about the parental/caregiver role and the need to strengthen awareness about quality time spent together.

*“Then there’s also how do you get the parents [to understand] how important it is. It really isn’t about quality of time, it’s about quantity, as well. You know, being there. How do we get back to that message?” (SC 9)*

Participants discussed the importance of creating connections between families and how this enhances opportunities for free play and independent mobility. They highlighted that these norms had existed in the past and should be brought back.

So, I think some of the older people in the community saw that this was something that occurred when they were young. They could remember back when they were young that there were these groups and you went back and forth on playdates and stuff. Because of society [today], where you go to the arena or you can go to the dance hall or you can go to the auditorium, you have to go someplace. There’s not as much people coming home after school and playing like four or five kids. Like when I was young, 4 or 5 of us would end up coming to my place and we’d hang out in the backyard or something like that. It was that kind of localized thing. (SC 2)

I think Planet Youth will really help to bolster a sense of community, and just bring back more kind of trust in our kids that they will make the right decisions and not put themselves in too much danger. And be able to meet up with friends at the nearby park or whatever. Obviously not, you know, four-year-olds, but once they get to be like ten or 11, you know? More freedom. And it’s interesting, they’ve done studies and the area that [children] can travel to has gotten so much smaller over the years. So, it used to be that they could go like 5 km you know, once they hit a certain age like 7-9 kind of thing. They could walk or bike or whatever and then gradually getting smaller and smaller and smaller. Now, I think it’s ridiculous like its 20 meters or something. It’s pretty intense. So just the change. (SC 3)

#### Opportunities presented by the pandemic

The last theme re-visits many of the issues identified in the other themes that relate to daily life before the pandemic and explores how community members perceived the pandemic influenced these factors. Interviews were completed in the first few months of the global COVID-19 pandemic. Therefore, Steering Committee members were able to share their experiences of the changes in their communities as well as how they were influencing social norms and daily life. Several participants identified how public health restrictions influenced family life and the amount of time families spent together … *“there’s so many people that I’ve heard of on Facebook and things like that are saying, you know such blessings that they’ve had, by being with their families, that this opened their eyes to a lot of things.” (SC 9)*

Part of it too is that people had to reconnect with their families so kids and parents will play more board games and are more encouraged to hang out together and parents finally have the time to bake cookies with their kids. You know, like I think a lot of that relationship building has been really helpful. (SC 3)

In addition, it created a blending between home and school contexts, where parents and caregiver became integrated in classroom experiences and gained insight into their children’s educational experiences.

We’ve been forced through COVID to spend time with our kids. And I mean just think about how many parents, you included, have sat there with their kids online doing schoolwork. That didn’t happen 6 months ago. It was like, ok, here’s your computer, go and do your work, you know, that was it and like when they came downstairs, it was like “did you finish?” “yeah” and that was it. (SC 2)

One participant also identified how pandemic work-from-home policies have impacted family schedules through the elimination of longer commutes. This is particularly relevant for families living in a rural context that live further away from their work locations in urban centres.

I’m in a place where I can’t come and pick you up, if you want to stay for an hour or two and do some sports, well you have to leave the school and go to the youth centre, because they’ll stay open until 6 or 7. You know, and so that’s the dynamic of, you know, we’re next to an urban centre where people work. People don’t get back to the community until 5:30 or 6:00. That, again, has changed with COVID because a lot of people aren’t doing that anymore. (SC 2)

One Steering Committee member remarked that he noticed an increased community presence as people were out walking more often, *“Over the last several months, people are out and about more than they used to be, I think. Out walking.” (SC 1)* Another participant noted that mental health referrals were down and suspected that this might be a reflection of reduced stress related to taking a break from school.

So, a lot of our Mental Health Partners will tell you that throughout COVID, the numbers dropped significantly of the referrals that they were getting. So that could be that there’s a decrease in stress from school, right? Which can be a really stressful place for kids. Especially if there’s anything like bullying or anything like that going on. But just the schoolwork itself, you know, can be stressful. (SC 3)

Finally, one Steering Committee member also identified that the pandemic health restrictions has forced society to alter daily behaviour in significant ways and that this might create increased adaptability and willingness to change in response to planned IPM interventions.

Interestingly enough, [the community] may be more ready for a change after COVID … because people have had to change, because they have altered the way they live. Maybe there’s an opportunity for there to be more interest. So, when we come up and say, here’s the results, this is what’s been happening with youth in the community. People might say, well yeah, we need to change that because they’ve already been forced to go through some significant change in the last 6 months. (SC 2)

## Discussion

This paper applies the bioecological model to examine substance use behaviours in youth that are influenced by time use patterns and context-related factors. We also explore how the pandemic influenced these issues. With respect to the bio-ecological model, the first theme describes the inter-relationships between the concepts of time and space, while the second theme also includes considerations related to the family micro-system and with respect to the development of supportive connections within meso-systems. The third theme illustrates how the pandemic has influenced each of the aforementioned components of the bioecological model, including time and space use, the family microsystem and interconnections among meso-systems.

One of the main concerns that were raised by Steering Committee members was how the excess of unstructured after school time and the challenges created through a lack of transportation and accessibility of programming contributed to existing challenges with youth substance use. Unstructured unsupervised time spent with peers is associated with substance use behaviours in youth (Hoeben et al., 2016; Meldrum & Leimberg, 2018; Osgood et al., 1996). Based on the February 2022 school-wide survey results, young people who were out past midnight once or more in the past week were more likely to smoke, use cannabis and to have been drunk in the past 30 days (Planet Youth Lanark County, 2022). Further, through a youth participatory asset-mapping that was completed in the spring of 2022, young people identified that extracurricular opportunities were limited and they have nothing to do (Planet Youth Lanark County, 2022).

Interestingly, participants also noted that the lockdowns and remote work that were enforced during this time period influenced the after-school time period, in that many parents were no longer spending time in a lengthy commute. Studies in Iceland and Canada have identified that pandemic lockdowns were associated with a decline in youth substance use behaviours (Hawke et al., 2021; Thorisdottir et al., 2021). This may be the result of reduced time spent in unstructured unsupervised activities with peers as many families were spending the majority of their time at home, with each other. This outcome may not be entirely dissimilar to impacts identified in past IPM interventions where curfews, parent/caregiver presence in communities and increased time with family reduced substance use behaviours in young people (Sigfúsdóttir et al., 2009).

Remote work can enhance flexibility and promote work-life balance for parents and caregivers. Time lost during commutes can now be spent at home with family or managing household obligations. The pandemic placed a significant burden on women with setbacks on gender equality in the workplace (Carli, 2020; Fisher & Ryan, 2021; International Labour Organization, 2021; Power, 2020; Wade et al., 2021). However, the pandemic also brought attention to the need for key family supports to reduce the disproportionate domestic workload placed on women (Power, 2020). In Canada, several recent policies were developed that can alleviate the care burden placed on women through increased support for sick pay and day care (see Government of Canada, 2021a; Ontario, 2021). Internationally, recent pilots of successful 4-day workweeks can also support family work-life balance, productivity, wellbeing as well reduced emissions (Fioramonti et al., 2022; Fitzgerald, 2022).

The new work-from-home paradigm not only impacts family schedules, it also has significant employment implications for young people living in rural context. Since many jobs are now accessible through remote working, there are now more diverse employment opportunities that are available to young people living in rural communities. Rather than choosing to leave their home communities for work, they may now have the option to stay. This will likely enhance career development opportunities for local youth, benefit the economy, and strengthen community connections.

Participants identified that they hoped the IPM intervention would strengthen community connections and enhance opportunities for children’s independent mobility. It is interesting to note that other research has identified that the pandemic has influenced these behaviours in younger children (Pelletier et al., 2021). During lockdowns, most recreational activities were unavailable while parents/caregivers were forced to provide childcare during their work hours which led to circumstances where children experienced an increase in independent mobility and unstructured play (Pelletier et al., 2021). Encounters with risky play are made possible when children have the time, space, and freedom in their day for unstructured play (Brussoni et al., 2015; Tremblay et al., 2015). As children’s access to independent mobility increases, it may also enhance capacity for assessment and management of risk to support healthy decision-making in adolescence with respect to substance use (Ungar, 2007).

Another key finding that was communicated by the participants was the need for stronger connections, both among family members, between families and among neighbours. Within the bioecological model, community connections would be represented by meso-systems and strengthening community connections represents a key strategy that is often used in the IPM. Some participants also observed changes that resulted from the pandemic that can impact these connections, such as increased time with family and more time spent outdoors in the community. These findings have been identified in other studies, including increased quality time with family (Evans et al., 2020; Pelletier et al., 2021; Williams et al., 2021), and increased family bonding (Evans et al., 2020; Pelletier et al., 2021; Power, 2020). Research has also identified that there was decreased bullying in schools during the pandemic (Vaillancourt et al., 2021) and a reduction in stress related to the fast pace of life and pressure from school (Cornell, 2021; Hawke et al., 2020; Power, 2020). Many of these changes are related to time use patterns and the hectic pace of life. The Canadian Index of Wellbeing includes a Time Use Domain that captures indicators focused on time spent at work, time spent with friends, commute time and experience of time pressure (Canadian Index of Wellbeing, 2021). Further, research using the index has found that increased time spent commuting is associated with an increased sense of time pressure and reduced life satisfaction (Hilbrecht et al., 2014).

We also collectively spent more time outdoors during the pandemic (Pelletier et al., 2021; Williams et al., 2021), and across the world, policies were created to reallocate roads to facilitate social distancing, active travel and outdoor recreation (Combs & Pardo, 2021). This shift presents a significant opportunity to support social justice and to promote outdoor play opportunities for children (Russell & Stenning, 2020). Spending more time in our local neighbourhoods can strengthen community connections (Innocent & Stevens, 2021) and make neighbourhoods safer (McMillen et al., 2019; Rydin et al., 2012; Saville, 2009). These strategies align with the IPM approach and may have a positive influence on substance use behaviours in young people.

Finally, participants noted that significant shifts in social norms that resulted from the pandemic may impact community willingness to change. This is an important factor in adopting new interventions (Halsall, et al., forthcoming) and is particularly important in the case of the IPM as communities often experience initial difficulties building stakeholder buy-in (Bajwa, 2017).

### Limitations and future research

Although, we have pursued efforts for broader commnity youth engagement in this research, the COVID-19 pandemic has made this difficult. Schools in the province were locked down for a total of 28 weeks and public health representatives on the youth engagement committee were repeatedly drawn into COVID work. Since the spring of this year, we have successfully captured more young people’s perspective through an asset mapping that is now published online (see: Planet Youth Lanark County, 2022) and we have begun to implement community consultations with youth. These will help to guide the development of community-level strategies to support substance use prevention as well as future research priorities.

We present an exploratory study that took advantage of key insights that were derived within a community that is implementing the IPM during the early stages of the pandemic. These experiences would be unique to the particular context, however, the phenomena observed have relevance for the implementation of collaborative interventions that apply an ecological approach to support youth development and wellbeing. “Research on systems needs to provide policy makers and practitioners with robust and relevant evidence that takes adequate account of the real-world circumstances in which people live, policies are made, and interventions are implemented.” (Rutter et al., 2017, p. 2602). Using the concepts of time and space as a framework, this paper examines system-level changes to organize key issues within youth substance use prevention. Future research should continue to examine how comprehensive initiatives that influence time and space use may impact youth substance use and overall development.

In particular, the relationship between unstructured time, developmental stage, risky play and later involvement in substance use requires additional study. The “optimal” amount of unstructured time may change as a function of age with reasonable levels during childhood to support opportunities to experience risk management and cognitive development and then moderated as individuals move into adolescence to avoid boredom and to promote constructive use of time. Future research should examine the boundaries of age where unstructured time no longer contributes to enhancement of cognitive risk assessment and a reduction in later problematic involvement in substance use. Similarly, research should examine whether unsupervised time in adolescence remains a risk factor if individuals experience significant opportunities for free and risky play in their childhood.

Free and unstructured play also typically necessitates low adult supervision, yet it is in these scenarios that bullying can become problematic. Recent research found that under the public health restrictions of the pandemic, bullying incidents decreased, possibly resulting from increased supervision at schools (Vaillancourt et al., 2021). Therefore, it is important to better understand what conditions are necessary to promote free and risky play while also mitigating the risks of bullying.

Future research should pursue broader youth engagement both in intervention and research design to integrate their lived experience of key equity issues in decision-making to help focus efforts on risk and protective factors that influence developmental contexts. Integrating young people in this work will help to enhance community engagement in health promotion and upstream efforts (Halsall et al., forthcoming).

This research placed a focus on opportunities presented by the pandemic, however most research studies found a decline in child and youth mental health during the pandemic (Children First Canada, 2022). Although it is not clear how mental health was affected, researchers have suggested that social isolation, academic challenges, increased exposure to screens and lack of access to services and extra-curricular activities may be factors that were involved (Allison et al., 2022; Children First Canada, 2022; Spettigue et al., 2021). There is accumulating research that has identified that social media and screen time is associated with poor mental health (Abi-Jaoude et al., 2020; Domingues‐Montanari, 2017; Khan et al., 2021). Ecological interventions with a policy orientation, such as the IPM, have the potential to mitigate the negative effects of screen time on youth mental health and should be a priority focus for both implementation and research in the future. These efforts can support the creation of healthier environments for young people and to reduce the strain on the mental health system so that those who need healthcare, will receive it.

## Conclusion

This paper offered a conceptual review of research examining youth substance use prevention through the lens of the bioecological model with particular consideration of time and space use interactions. It also applies these concepts within a case study of the implementation of PYLC. In addition, we reflect on the multitude of effects that were created by the COVID-19 pandemic that highlight interactions between massive social change and how this might influence child and youth development. Future research should continue to examine how these changes influence child and youth development and they should be applied in future policy and practice to support healthy child and youth development.

## Data Availability

The data that support the findings of this study are available on request from the corresponding author. The data are not publicly available due to privacy or ethical restrictions.
